# Supplementary material for: Modeling glioblastoma heterogeneity as a dynamic network of cell states
Source: Mol Syst Biol. 2021 Sep 16;17(9):e10105. doi: 10.15252/msb.202010105 (PMC8444284; doi:10.15252/msb.202010105)
Supplement: Supplementary file 6 — Source Data for Figure 5 [file MSB-17-e10105-s004.zip › Figure5A_sourcedata/GSEA_3071/nosigdb_stateI.GseaPreranked.1622795145664/NB_KI_CLUSTER13.html]

Details for gene set NB\_KI\_CLUSTER13[GSEA]

|  || Dataset | state13071 |
| Phenotype | NoPhenotypeAvailable |
| Upregulated in class | na\_pos |
| GeneSet | NB\_KI\_CLUSTER13 |
| Enrichment Score (ES) | 0.43022943 |
| Normalized Enrichment Score (NES) | 2.440442 |
| Nominal p-value | 0.0 |
| FDR q-value | 0.0 |
| FWER p-Value | 0.0 |
Table: GSEA Results Summary

  

Fig 1: Enrichment plot: NB\_KI\_CLUSTER13      
 Profile of the Running ES Score & Positions of GeneSet Members on the Rank Ordered List

  

| PROBE | GENE SYMBOL | GENE\_TITLE | RANK IN GENE LIST | RANK METRIC SCORE | RUNNING ES | CORE ENRICHMENT || 1 | LEF1 |  |  | 102 | 1.084 | -0.0033 | Yes |
| 2 | FAM129A |  |  | 135 | 0.953 | 0.0106 | Yes |
| 3 | LSP1 |  |  | 143 | 0.930 | 0.0313 | Yes |
| 4 | NABP1 |  |  | 154 | 0.907 | 0.0505 | Yes |
| 5 | ANKRD28 |  |  | 173 | 0.865 | 0.0663 | Yes |
| 6 | APOL6 |  |  | 178 | 0.859 | 0.0861 | Yes |
| 7 | TNFAIP8 |  |  | 179 | 0.858 | 0.1070 | Yes |
| 8 | SAMD9L |  |  | 180 | 0.857 | 0.1279 | Yes |
| 9 | XBP1 |  |  | 215 | 0.797 | 0.1375 | Yes |
| 10 | MAF |  |  | 227 | 0.773 | 0.1531 | Yes |
| 11 | TAPBP |  |  | 287 | 0.692 | 0.1528 | Yes |
| 12 | SOCS1 |  |  | 292 | 0.686 | 0.1684 | Yes |
| 13 | CAST |  |  | 298 | 0.675 | 0.1834 | Yes |
| 14 | GBP3 |  |  | 316 | 0.658 | 0.1945 | Yes |
| 15 | SH3KBP1 |  |  | 340 | 0.636 | 0.2033 | Yes |
| 16 | TRIM22 |  |  | 348 | 0.630 | 0.2166 | Yes |
| 17 | BTN3A2 |  |  | 366 | 0.624 | 0.2269 | Yes |
| 18 | TRIM38 |  |  | 386 | 0.602 | 0.2360 | Yes |
| 19 | MDFIC |  |  | 389 | 0.601 | 0.2501 | Yes |
| 20 | EPSTI1 |  |  | 394 | 0.592 | 0.2633 | Yes |
| 21 | TNFSF10 |  |  | 395 | 0.590 | 0.2777 | Yes |
| 22 | SQSTM1 |  |  | 412 | 0.578 | 0.2872 | Yes |
| 23 | IFI44L |  |  | 422 | 0.574 | 0.2985 | Yes |
| 24 | HERPUD1 |  |  | 436 | 0.561 | 0.3085 | Yes |
| 25 | LY96 |  |  | 451 | 0.547 | 0.3177 | Yes |
| 26 | NAMPT |  |  | 456 | 0.544 | 0.3298 | Yes |
| 27 | SSR4 |  |  | 459 | 0.543 | 0.3425 | Yes |
| 28 | DDX60 |  |  | 481 | 0.527 | 0.3492 | Yes |
| 29 | SKAP2 |  |  | 483 | 0.526 | 0.3618 | Yes |
| 30 | ARHGAP18 |  |  | 501 | 0.510 | 0.3692 | Yes |
| 31 | RSRP1 |  |  | 537 | 0.488 | 0.3709 | Yes |
| 32 | PIEZO1 |  |  | 567 | 0.471 | 0.3740 | Yes |
| 33 | IFI35 |  |  | 574 | 0.467 | 0.3836 | Yes |
| 34 | TCEA3 |  |  | 633 | 0.440 | 0.3774 | Yes |
| 35 | KLF6 |  |  | 647 | 0.433 | 0.3842 | Yes |
| 36 | PSMB8 |  |  | 669 | 0.423 | 0.3884 | Yes |
| 37 | GBP2 |  |  | 674 | 0.422 | 0.3975 | Yes |
| 38 | AGTRAP |  |  | 676 | 0.422 | 0.4075 | Yes |
| 39 | VDR |  |  | 681 | 0.420 | 0.4166 | Yes |
| 40 | MYADM |  |  | 697 | 0.413 | 0.4223 | Yes |
| 41 | BHLHE40 |  |  | 720 | 0.405 | 0.4258 | Yes |
| 42 | DUSP1 |  |  | 787 | 0.386 | 0.4160 | Yes |
| 43 | OSTF1 |  |  | 808 | 0.378 | 0.4194 | Yes |
| 44 | SLC40A1 |  |  | 848 | 0.366 | 0.4169 | Yes |
| 45 | PQLC3 |  |  | 852 | 0.364 | 0.4249 | Yes |
| 46 | DDIT4 |  |  | 865 | 0.361 | 0.4302 | Yes |
| 47 | SERPINB1 |  |  | 918 | 0.346 | 0.4235 | No |
| 48 | IFIH1 |  |  | 962 | 0.336 | 0.4192 | No |
| 49 | PARP14 |  |  | 1026 | 0.323 | 0.4087 | No |
| 50 | RNF213 |  |  | 1027 | 0.322 | 0.4166 | No |
| 51 | CTSA |  |  | 1065 | 0.314 | 0.4134 | No |
| 52 | TNFRSF14 |  |  | 1109 | 0.305 | 0.4083 | No |
| 53 | APOL3 |  |  | 1128 | 0.302 | 0.4105 | No |
| 54 | MX1 |  |  | 1130 | 0.302 | 0.4175 | No |
| 55 | NR3C1 |  |  | 1162 | 0.297 | 0.4157 | No |
| 56 | TAP2 |  |  | 1171 | 0.295 | 0.4206 | No |
| 57 | CAPG |  |  | 1222 | 0.285 | 0.4130 | No |
| 58 | REST |  |  | 1225 | 0.285 | 0.4193 | No |
| 59 | TMBIM1 |  |  | 1339 | 0.261 | 0.3928 | No |
| 60 | RAP1B |  |  | 1400 | 0.252 | 0.3815 | No |
| 61 | CD44 |  |  | 1430 | -0.251 | 0.3791 | No |
| 62 | TBC1D1 |  |  | 1674 | -0.277 | 0.3151 | No |
| 63 | EHD4 |  |  | 1726 | -0.283 | 0.3071 | No |
| 64 | SELENOK |  |  | 2067 | -0.328 | 0.2161 | No |
| 65 | ETS1 |  |  | 2086 | -0.332 | 0.2190 | No |
| 66 | CAVIN2 |  |  | 2237 | -0.360 | 0.1841 | No |
| 67 | CALCRL |  |  | 2249 | -0.363 | 0.1897 | No |
| 68 | PTPRJ |  |  | 2312 | -0.374 | 0.1808 | No |
| 69 | BTG2 |  |  | 2318 | -0.375 | 0.1885 | No |
| 70 | AC016831.1 |  |  | 2461 | -0.404 | 0.1569 | No |
| 71 | JUND |  |  | 2670 | -0.464 | 0.1077 | No |
| 72 | HOXB3 |  |  | 2735 | -0.482 | 0.1008 | No |
| 73 | SMIM3 |  |  | 2783 | -0.498 | 0.0992 | No |
| 74 | ARHGEF6 |  |  | 2799 | -0.501 | 0.1071 | No |
| 75 | ITPR2 |  |  | 3002 | -0.603 | 0.0629 | No |
| 76 | CCDC85B |  |  | 3075 | -0.657 | 0.0580 | No |
| 77 | DUSP6 |  |  | 3227 | -0.816 | 0.0339 | No |
| 78 | CD82 |  |  | 3261 | -0.870 | 0.0455 | No |
| 79 | ID2 |  |  | 3374 | -1.110 | 0.0399 | No |
Table: GSEA details [plain text format]

  

Fig 2: NB\_KI\_CLUSTER13: Random ES distribution      
 Gene set null distribution of ES for **NB\_KI\_CLUSTER13**
